# Factors affecting medical file documentation during telephone triage at an emergency call centre: a cross-sectional study of out-of-hours home visits by general practitioners in France

**DOI:** 10.1186/s12913-019-4350-4

**Published:** 2019-07-30

**Authors:** Matthieu Heidet, Florence Canoui-Poitrine, François Revaux, Thomas Perennou, Maeva Bertin, Charles Binetruy, Julien Palazzi, Eric Tapiero, Michel Nguyen, Paul-Georges Reuter, Eric Lecarpentier, Julien Vaux, Jean Marty

**Affiliations:** 1 Assistance Publique - Hôpitaux de Paris (AP-HP), Hôpital Universitaire Henri Mondor, SAMU 94 et Urgences, Créteil, France; 2 Université Paris-Est Créteil (UPEC), EA-4390 (Analysis of Risk in Complex Health Systems, ARCHeS), Créteil, France; 3 Assistance Publique - Hôpitaux de Paris (AP-HP), Hôpital Universitaire Henri Mondor, Département de Santé Publique, Créteil, France; 4 Université Paris-Est Créteil (UPEC), EA-7376 (Clinical Epidemiology and Ageing, CEpiA), Créteil, France; 5 Assistance Publique - Hôpitaux de Paris (AP-HP), Hôpital Universitaire Raymond Poincaré, SAMU 92, Garches, France

**Keywords:** Emergency call Centre, Telephone triage, Out-of-hours primary care, Organization, Quality

## Abstract

**Background:**

In France, general practitioners (GPs) perform out-of-hours home visits (OOH-HVs) after physician-led telephone triage at the emergency call centre. The quality of a systematic physician-led triage has not been determined in France and may affect the efficiency of the OOH-HV process. The objectives of this study were first, to evaluate the quality of reporting in the electronic patient’s file after such triage and second, to analyse the factors associated with altered reporting.

**Methods:**

Cross-sectional study in a French urban emergency call centre (district of Paris area) from January to December 2015. For a random selection of 30 days, data were collected from electronic medical files that ended with an OOH-HV decision. Missing key quality criteria (medical interrogation, diagnostic hypothesis or ruled-out severity criteria) were analysed by univariate then multivariate logistic regression, adjusted on patient, temporal and organizational data.

**Results:**

Among 10,284 OOH-HVs performed in 2015, 748 medical files were selected. Reasons for the encounter were digestive tract symptoms (22%), fever (19%), ear nose and throat symptoms, and cardiovascular and respiratory problems (6% each). Medical interrogation was not reported in 2% of files (*n* = 16/748) and a diagnostic hypothesis in 58% (*n* = 432/748); ruled-out severity criteria were not reported in 60% (*n* = 449/748). On multivariate analysis, altered reporting was related to the work overload of triage assistants (number of incoming calls, call duration, telephone occupation rate; *p* < 0.03).

**Conclusion:**

In the electronic files of patients requiring an OOH-HV by a GP in a French urban area, quality in medical reporting appeared to depend on organizational factors only, especially the triage assistants-related work factors. Corrective measures are needed to ensure good quality of triage and care.

**Electronic supplementary material:**

The online version of this article (10.1186/s12913-019-4350-4) contains supplementary material, which is available to authorized users.

## Background

In western countries, the demand for same-day access to out-of-hours primary care (OOH-PC) continues to increase [1]. In France, public emergency call centres receive all health-related incoming calls and coordinate access to OOH-PC providers, including mobile general practitioners (GPs) performing semi-urgent OOH home visits (OOH-HVs; i.e., visits that cannot wait until the reopening of primary care practices). Some clinical situations warrant a home visit by a GP, because of the need for a rapid clinical assessment or treatment or because of the impact of an emergency department referral, especially in frail and older patients [2]. A safe and qualitative access to OOH-PC represents a major objective for health systems that have to deal with emergency department overuse [3] and non-optimal efficiency [4].

In French public emergency call centres, the call handling is two-tiered: first, non-medical assistants collect the initial information and can provide basic support. Then, a physician provides medical interrogation and decides on the most appropriate response. The French model of triage for access to OOH-PC is based on national recommendations [5]. Despite lack of high-level evidence, physician-led triage is believed to be a good model for the triage process, patient access to healthcare structures, safety and costs [6].

However, unlike in other countries and settings [1, 7–9], we lack studies analysing the triage process and quality of the data in the electronic file recorded during physician-led telephone triage leading to an OOH-HV in France. Thus, our aims were first, to describe the level of reporting of triage key indicators in the caller’s medical file before the OOH-HV decision in France, and second, to analyse factors associated with the absence of such indicators.

## Methods

### Design and setting

This was a cross-sectional study of patients calling the medical emergency call centre of Créteil (Henri-Mondor hospital, district of Val-de-Marne, Paris area) between January and December 2015 and for whom an OOH-HV was prescribed.

The OOH-PC system runs between 8:00 pm and 8:00 am on weekdays, 12:00 am and 8:00 am on Saturdays and 24 h on Sundays and public holidays. The system provides for medical contact any time it is needed and is covered by social insurance. Public emergency call centres exist to handle every health-related incoming call. After an initial handling by non-medical assistants, physicians such as GPs or emergency physicians (EPs) proceed to medical interrogation and decide on the best response. They complete a three-tiered electronic medical record: medical interrogation (free text), codification of the reason for the call and/or reason for encounter (RFE; by the *International Statistical Classification of Diseases and Related Health Problems*, 10th Revision [ICD-10]), and codification of the decision taken (assisted). The triage GPs at the emergency call centre are employees of private general practices commissioned by the hospital hosting the call centre. GPs performing the OOH-HV are members of the latter practices and often have knowledge of telephone triage. Triage EPs are employees of the hospital hosting the call centre.

### Data source and collection

#### Database

We used the database of the electronic patient administration system of the Val-de-Marne emergency call centre during 2015 (Centaure 15; SIS, France). Every incoming call generates an electronic medical file, identified by a unique number. All collected data are then linked to this file: the patient’s identity and medical history, type of physician performing the triage, interrogation, classification of the RFE, and final decision.

#### Sample selection

In accordance with recommendations of the French ministry of health (HAS) [5] and with the high volume of eligible files (*n* = 10,284), we used a weighted and stratified random sample of data from 30 non-consecutive days of the entire year 2015. We had two main reasons for choosing a 30-day period: first, 30 days seemed like an acknowledgeable and convenient time unit, and second, because of restrained logistics (staff size, time constraints, need for manual data-mining [see infra]), the amount of eligible files was acceptable [10].

To ensure good representativeness of the sample, the 30-day selection was stratified on the determinants of incoming calls (factors known or suspected to be associated with increased demand for OOH-PC and/or OOH-HV, such as epidemics, seasons, holidays). To this end, an expert panel of physicians regularly performing telephone triage in France was created; this panel followed a RAND/UCLA appropriateness method (RAM) [11] to achieve consensus on the main determinants to be sampled. In a two-round process, experts used a scale from 1 to 9 to rate each determinant we proposed. After round one, experts were given the opportunity to propose other determinants to be rated in round two. Determinants with median scores ≥7 after round 2 were used for our model (see Additional file 1).

Experts were physicians working in four different French emergency call centres located in the Paris area. Some were EPs and others were GPs. All shared their professional activity between emergency call centres and other services (prehospital care, emergency departments, general practice, OOH-HV, etc.).

For the 30-day selection, strata during holidays or public holidays were over-sampled, with a sampling fraction of 20% (6 days), mainly because the OOH-PC system aims to ensure access during non-weekdays and/or non-daytime hours. The sampling fraction for other strata during the 24 remaining selected days was 5% (with each selected day containing at least one determinant, with possible combinations of several determinants).

#### File extraction

Medical files were extracted on the basis of the final decision of the OOH-HV. To limit the inaccuracy of ICD-10 diagnostic codes (which could change from the code for an “infant with fever and rhinitis” to that for “paediatrics”, therefore leading to potentially numerous diagnoses for similar cases), manual data mining was performed to refine the RFE, which were then classified according to the specific *International Classification for Primary Care,* Second Edition code (ICPC-2) [12]. The manual data mining consisted of systematically re-reading every file, especially the free text the physician had typed, to homogenize the final ICD-10 diagnostic codes into ICPC-2 codes. For example, when an ICD-10 code was “paediatrics”, the ICPC-2 code was implemented depending on the specific symptoms and/or diagnoses documented in the free text space (“fever”, “diarrhea” or “otitis”).

To analyse the impact of organizational factors on the quality of the medical file, monitoring data for the call centre were extracted: workforce at the time of the call (number of working assistants or physicians during a shift of 8 h), call duration and rate of telephone occupation of assistants and physicians (minutes spent on phone during a working shift of 8 h, and percentage of total occupation time), and number of mobile GPs working at the time of the call.

### Endpoints

The endpoints were the absence of the key indicators in the medical file: medical interrogation, suspected diagnoses, and ruled-out severity.

### Variables

Data extracted from the medical file were the day and time of the call (weekday, weekend, public holiday, holiday, 00:00–04:00 am, 04:00–08:00 am, 08.00–00.00 am) patient characteristics (age (< 15, 15–64, > 65 years), sex, RFE (see Table 1), medical interrogation, diagnostic hypothesis, ruled-out criteria for severity (i.e., if at least one negative sign was reported, such as “no chest pain”, “no dyspnea”, etc., severity criteria was considered reported), diagnostic code and decision after a face-to-face encounter (remain at home, referred to the hospital).

**Table 1:** Reasons for OOH-HV encounter (after systematic review of medical files and recodification according to the *International Classification for Primary Care, Second Edition* [ICPC-2]) (*n* = 748)

RFE	ICPC-2 code	N	%	95% CI
Digestive tract problem		163	22	[13–19]
Abdominal pain	D01–03;D06	84	11	[9–12, 20, 21]
Vomiting	D10	52	7	[5–9]
Diarrhea	D11	27	4	[2–5]
Fever	A03	141	19	[13–16, 22–24]
ENT problem		49	6	[5–9]
Cardiovascular		49	6	[5–9]
Chest pain	A11	26	3	[2–5]
Palpitations	K04	12	1	[1–3]
Hypertension	K86,KB7	11	2	[1–3]
Respiratory problem		45	6	[4–8]
Cough		25	3	[2–5]
Short breath/dyspnea		20	3	[2–4]
Dorsolumbar problem		44	6	[4–8]
Without irradiation	L84	43	6	[4–8]
With irradiation	L86	1	< 1	–
Malaise		41	5	[4–7]
Fainting/syncope	A06	35	5	[3–6]
Asthenia	A04	6	1	[0–2]
Missing data		216	29	[25–31]

*OOH-HV* out-of-hours home visits, *RFE* reasons for encounter, *ENT* ear-nose-throat, *95% CI* 95% confidence interval

### Data management and analysis

We performed a descriptive analysis of the RFE (by age class, date and time of call) and key indicators in the electronic medical file. Categorical data are reported as number (%) with 95% confidence intervals (CIs) and continuous data as mean ± SD or median (interquartile range [IQR]). After testing for linearity gap, continuous data were categorized for analysis. We used three univariate analysis rounds, one for each endpoint (unreported medical interrogation, diagnostic hypothesis, ruled-out severity criteria), by using logistic regression.

For multivariate analysis, odds ratios (ORs) are reported with 95% CIs. Multivariate regression models were adjusted for variables associated with *p* < 0.2 on univariate analysis and variables known or suspected to be associated with the endpoints, such as patient characteristics (age, sex, type of caller [patient, bystander, caregiver, undetermined]) and contextual variables (day and hour of call). Because the main objective of the OOH-HV is to ensure access to care when general practices are closed, the analysis was adjusted on every variable related to the day and time of call. After testing for colinearity within the same group of variables, every variable related to the emergency call centre was tested in the multivariate analysis. As for univariate analysis, we produced three multivariate models of logistic regression (one for each studied endpoint).

All tests were two-tailed. *P* < 0.05 was considered statistically significant. All analyses involved using Stata 13 (StataCorp, College Station, TX).

## Results

### Factors of stratification

After applying the RAM to achieve consensus on the main strata, to be sampled, the expert panel identified flu epidemic, bronchiolitis, heat wave, pollution peak, and holidays and public holidays, for 10 determinants (or factors) of stratification. Each strata could contain several determinants (“heat wave” occurring during summer “holidays”) (see Additional file 1).

### Population

**Fig. 1:**
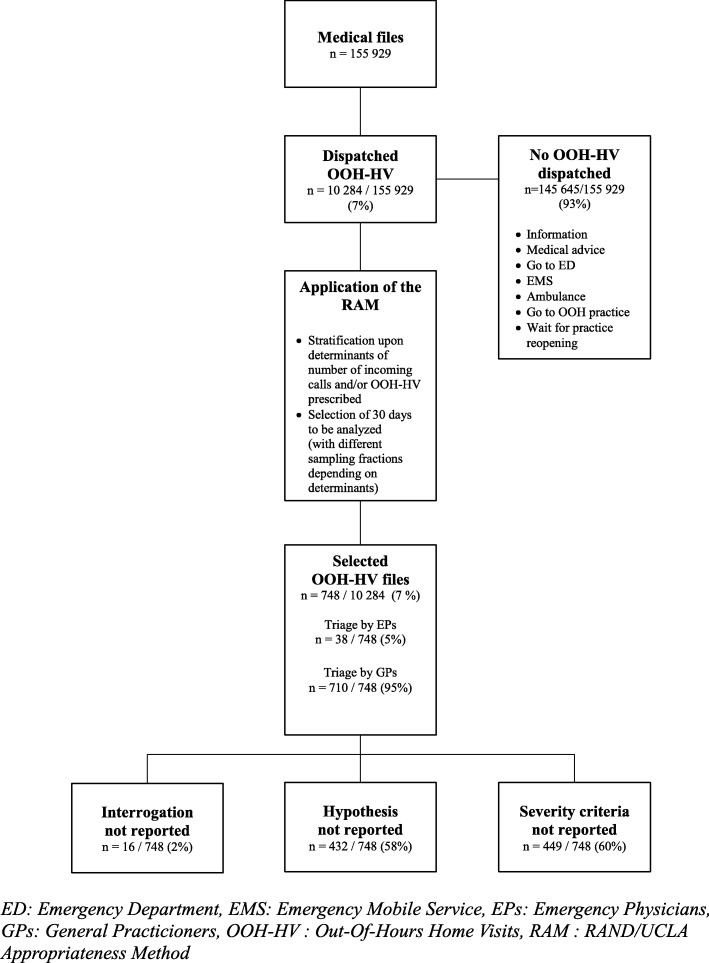
Flowchart of files analysed (from all health-related calls incoming to the emergency call centre)

Among the 10,284 OOH-HVs performed from January 1, 2015 to December 31, 2015, we selected for analysis 748 unique electronic medical files with an OOH-HV performed during the randomly selected 30 days (19 holiday or public holiday days, 2 weekend days and 9 weekdays of worked weeks) (Fig. 1). For 708 records (95%), medical triage was performed by GPs and for the other 5% by EPs.

In all, 455 callers were women (61%) and the median age was 33 [IQR 8–58]. When patients were children (age < 15), the median age was 2 [1–6]. Callers were largely bystanders (58%, n = 436) or patients themselves (31%, n = 231). Overall, 6% (n = 48) of the calls were from emergency first responders. Calls were predominantly received before midnight (61%, n = 455).

### Reasons for encounters

RFEs were digestive tract symptoms (22%, n = 163); fever (19%, n = 141); ear, nose and throat symptoms; cardiovascular, respiratory and dorsolumbar problems (6% each); and dizziness and fainting (5%, n = 41) (Table 1). RFEs were not reported in 29% of files (n = 216). After the OOH-HV, 8% of patients (n = 58) were referred to a hospital.

### Quality criteria not reported in medical files

Medical interrogation was not reported in 16/748 files (2% [95% CI 1.2–3.4]). The diagnostic hypothesis formulated by the triage GP was not reported in 432/748 files (58% [54–61]), and ruled-out severity criteria were not reported in 449/748 (40%) (Fig. 1). Only 14/748 files (2% [1.0–3.1]) had every quality criterion present, and 9/748 (1% [0.5–2.3]) had none of the criteria.

### Univariate analysis of factors associated with unreported criteria

For unreported medical interrogation, factors associated with altered quality of reporting were a call received during public holidays (reference weekday, OR 14.7 [2.9–73.2]), duration of call by triage assistants (reference ≤48 s, OR 1.1 [1.1–1.1]) and triage physicians’ telephone occupation rate (reference ≤33.7%, OR 0.5 [0.3–0.9]) (all p < 0.02) (Table 2). For unreported diagnostic hypothesis, factors were patient age < 15 years (OR 1.5 [1.0–2.1]), digestive RFE (reference general, OR 1.5 [1.1–2.3]), number of incoming calls to triage physicians (reference ≤9700, OR 0.8 [0.7–0.9]), call duration (triage assistants, reference ≤48 s, OR 1.1 [1.1–1.1]; physicians, reference ≤147 s, OR 1.2 [1.1–1.3]) and physicians’ telephone occupation rate (reference ≤33.7%, OR 0.8 [0.7–0.9]) (p < 0.05). For unreported ruled-out severity criteria, factors were the hour of call (reference 8:00–00:00 am, for 00:00–04:00 am, OR 0.6 [0.4–0.8]), weekend call (reference, weekday call, OR 0.5 [0.3–0.9]), workforce at the call centre (assistants, reference 3, OR 1.2 [1.1–1.4]), number of incoming calls (assistants, reference ≤30,000, OR 1.2 [1.1–1.3]; physicians, reference ≤9700, OR 1.2 [1.1–1.4]), assistants’ call duration (reference ≤47 s, OR 1.1 [1.1–1.1]) and telephone occupation rate (assistants, reference ≤26.2%, OR 1.3 [1.1–1.5]).

**Table 2:** Factors associated with not reporting interrogation, hypothesis and severity criteria in medical files (univariate analysis)

	Interrogation not reported		Hypothesis not reported		Severity not reported	
	*n* = 16/748		*n* = 432/748		*n* = 449/748	
	uOR [95% CI] ^x^	p ^+^	uOR [95% CI]^x^	p ^+^	uOR [95% CI]^x^	p ^+^
Patient characteristics						
Sex, reference male	1.3 [0.4–3.8]	ns	1.1	ns	0.9 [0.7–1.2]	ns
Age, years		ns		ns		ns
Infant < 1	0.9 [0.1–8.2]		0.8 [0.4–1.4]		0.8 [0.5–1.5]	
Child < 15	0.9 [0.2–3.5]		1.5 [1.0–2.1]		1.2 [0.8–1.7]	
Adult 15–64	Ref.		Ref.		Ref.	
Adult ≥65	1.1 [0.3–3.8]		1.3 [0.9–1.8]		1.3 [0.9–1.8]	
Caller		ns		ns		ns
Patient	Ref.		Ref.		Ref.	
Bystander	0.3 [0.1–1.0]		1.2 [0.8–1.6]		1.1 [0.8–1.5]	
Other	1.2 [0.3–4.8]		1.3 [0.8–2.3]		1.0 [0.6–1.8]	
RFE		ns		ns		ns
Cardiovascular	2.5 [0.3–21.5]		1.2 [0.5–2.8]		0.4 [0.2–0.9]	
Digestive	1.3 [0.3–5.1]		1.5 [1.1–2.3]		0.9 [0.6–1.4]	
General	(ref)		(ref)		(ref)	
Osteoarticular	0.9 [0.1–7.3]		0.8 [0.5–1.4]		1.3 [0.7–2.1]	
Respiratory	2.8 [0.5–14.2]		0.6 [0.3–1.1]		1.3 [0.7–2.4]	
Potentially severe RFE^a^	2.3 [0.6–8.2]	ns	0.8 [0.5–1.3]	ns	0.8 [0.5–1.3]	ns
Time and day						
Time of call		ns		ns		*
8:00–00:00 am	Ref.		Ref.		Ref.	
00:00–4:00 am	1.1 [0.3–3.3]		0.8 [0.6–1.2]		0.6 [0.4–0.8]	
4:00–8:00 am	0.8 [0.2–3.6]		1.1 [0.7–1.6]		0.8 [0.5–1.3]	
Day		*		ns		ns
Weekday	Ref.		Ref.		Ref.	
Weekend	0.8 [0.1–7.2]		1.1 [0.6–1.9]		0.5 [0.3–0.9]	
Public holiday	14.7 [2.9–73.2]		0.8 [0.3–2.2]		0.8 [0.3–2.3]	
Holiday	1.1 [0.3–3.8]		0.9 [0.7–1.3]		0.7 [0.5–1.1]	
Emergency call centre						
Workforce (triage)						
Assistants, n						
3	Ref.		Ref.		Ref.	
> 3	0.9 [0.6–1.4]	ns	0.9 [0.8–1.1]	ns	1.2 [1.1–1.4]	*
Workload						
Incoming calls (monthly), n						
*Assistants*						
≤ 30,000	Ref.		Ref.		Ref.	
> 30,000	0.8 [0.5–1.3]	ns	0.9 [0.8–1.1]	ns	1.2 [1.1–1.3]	*
*Physicians*						
≤ 9700	Ref.		Ref.		Ref.	
> 9700	0.8 [0.5–1.3]	ns	0.8 [0.7–0.9]	*	1.2 [1.1–1.4]	*
Call duration, s (mean)						
*Assistants*						
≤ 48	Ref.		Ref.		Ref.	
> 48	1.1 [1.1–1.1]	*	1.1 [1.1–1.1]	**	1.1 [1.1–1.1]	*
*Physicians*						
≤ 147	Ref.		Ref.		Ref.	
> 147	1.3 [0.8–2.5]	ns	1.2 [1.1–1.3]	*	1.1 [0.9–1.1]	ns
Telephone occupation rate, %						
*Assistants*						
≤ 26.1	Ref.		Ref.		Ref.	
> 26.1	0.9 [0.5–1.3]	ns	0.9 [0.8–1.1]	ns	1.3 [1.1–1.5]	**
*Physicians*						
≤ 33.7	Ref.		Ref.		Ref.	
> 33.7	0.5 [0.3–0.9]	*	0.8 [0.7–0.9]	*	1.1 [1.0–1.3]	ns
Field						
Mobile GPs (per 1-GP increase), n						
1	Ref.		Ref.		Ref.	
> 1	1.0 [0.6–1.6]	ns	1.1 [0.9–1.2]	ns	1.1 [1.0–1.3]	ns

*RFE* reasons for encounter, *uOR* unadjusted odds ratio, x: logistic regression, +: Wald test

^a^: cardiac (chest pain, palpitations, dizziness/fainting/syncope), respiratory (acute dyspnea) or neurological (fainting, seizures, acute headache, meningitis syndrome), ns: non significant

*: p < 0,05, **: p < 0,001

### Multivariate analysis

On multivariate analysis, unreported interrogation remained linked to assistants’ call duration (reference ≤47 s, OR 12.9 [2.3–72.5]), unreported diagnostic hypothesis to assistant’s call duration (OR 2.1 [1.2–3.7]), and unreported ruled-out severity criteria to assistant’s call duration (OR 1.8 [1.1–3.1]) and telephone occupation rate (reference ≤26.1, OR 1.8 [1.3–2.5]) (Table 3).

**Table 3:** Factors associated with not reporting interrogation, hypothesis and severity criteria in medical files (multivariable analysis, adjusted on patient’s characteristics, day and time of call, and RFE)

	Interrogation not reported		Hypothesis not reported		Severity not reported	
	*n* = 16/748		*n* = 432/748		*n* = 449/748	
	aOR [95% CI]^x^	p ^+^	aOR [95% CI]^x^	p ^+^	aOR [95% CI]^x^	p ^+^
Emergency call centre						
Workforce (triage), n						
*Assistants*						
3	Ref.		Ref.		Ref.	
> 3	0.6 [0.3–1.2]	ns	0.8 [0.7–1.0]	ns	1.2 [0.4–3.0]	ns
Workload						
Incoming calls (monthly), n						
*Assistants*						
≤ 30,000	Ref.		Ref.		Ref.	
> 30,000	1.5 [0.4–6.7]	ns	1.0 [0.7–1.4]	ns	1.3 [0.9–2.0]	ns
*Physicians*						
≤ 9700	Ref.		Ref.		Ref.	
> 9700	2.0 [0.5–8.0]	ns	0.8 [0.6–1.2]	ns	1.3 [1.1–1.5]	ns
Call duration, s (mean)						
*Assistants*						
≤ 48	Ref.		Ref.		Ref.	
> 48	12.9 [2.3–72.5]	*	2.1 [1.2–3.7]	*	1.8 [1.1–3.1]	*
*Physicians*						
≤ 147	Ref.		Ref.		Ref.	
> 147	2.29 [0.7–11.5]	ns	1.3 [0.9–1.9]	ns	1.0 [0.7–1.4]	ns
Telephone occupation rate, %						
*Assistants*						
≤ 26.1	Ref.		Ref.		Ref.	
> 26.1	1.7 [0.5–6.0]	ns	0.9 [0.7–1.3]	ns	1.8 [1.3–2.5]	**
*Physicians*						
≤ 33.7	Ref.		Ref.		Ref.	
> 33.7	0.3 [0.1–1.2]	ns	0.7 [0.5–1.1]	ns	1.1 [0.8–1.5]	ns

*aOR* adjusted odds ratio, x: logistic regression, +: Wald test

*RFE* reasons for encounter; *ns* non significant, *: p < 0,05, **: p < 0,001

## Discussion

### Summary

Only 2% of the medical files for patients receiving an OOH-HV after a call at a French urban emergency call centre included all analysed quality criteria. On univariate analysis, individual data, such as age, sex, or the RFE, were not responsible for poor reporting in files. Factors associated with incomplete reporting appeared to be organizational only (workforce, workload, time of call, telephone occupation rate) and on multivariate analysis, remained significant for non-medical triage assistants only.

### Comparison with existing literature

Regarding the RFE, our results were globally similar to other European settings [13]. Organizational factors, such as workload, are known to be negatively associated with the quality of a given procedure [14, 15] and should be anticipated to enhance the reliability of a given system. Our study suggests that only triage assistant-linked factors affect the medical reporting. Prolonged interrogation by an assistant may be due to initial difficulties (unstructured interrogation and/or lack of communication skills [16]) that are discussed with the GP during the call transfer. Also, the longer the assistant’s interrogation, the more information that may accumulate [17]. The GP may benefit from unrecorded information. This situation could explain why the GP’s workload and call occupation did not affect our endpoints: if the assistant presents an interrogation as particularly difficult, only the most important components may be recorded (severity, diagnostic hypothesis). Furthermore, as compared with non-physician staff, and possibly due to differences in communication structure and decision process, GPs may need shorter communication time in telephone triage to prescribe an OOH-HV [18].

Two Dutch studies reported poor results for quality of telephone triage [19, 20]: although clinical problems were quasi-constantly reported, less than 30% of mandatory questions were asked, personal situation and medical history were missing in more than half of the files, emergency was underestimated in 41% of the calls, data on home management and safety-net advice were reported in 40% of patient files, clinical evaluation was imperfect, and triage outcome was appropriate for only 58% of the patients. Moreover, some unasked mandatory questions were still reported. Nonetheless, one of these studies focused on triage assistants, and calls were made by incognito-trained laypersons. Similar to our findings, individual data, including RFE, were not associated with altered results, which suggests that the system is equitable.

French data on public telephone triage are scarce and mostly focused on the impact of a single event on emergency call centre activity [21]. A recent monocentric study analysed the key performance indicators of an emergency call centre. The most important factor affecting answer time (< 20 s) was the overall telephone occupation rate [22]. This study showed significant temporal trends for the number of incoming calls, especially between 20:00 and 00:00 am. Our results showed the same evening trend in number of calls (61% incoming before midnight).

In Europe, telephone triage is not standardized [23]. Systematic physician-led triage is not supported by high-level evidence and has not been evaluated in terms of quality of reporting in the patient’s medical file but rather safety and efficacy [24–26]. GP-led triage, despite differences in decision-making process and information gathering [27] and longer training and experience as compared with nurses, showed similar safety and efficacy results. However, GPs could be more efficient in complex triage situations [28]. An optimal triage team could involve both staff types (nurses and GPs) [29]. The quality and safety of telephone triage remains an ongoing issue needing to be evaluated in methodologically robust prospective studies [30].

### Strengths and limitations

This is the first study to evaluate the typology of OOH-HVs by French GPs and the quality of medical files after telephone triage in an emergency call centre. In addition, it is the first time in France that the quality of information reporting in the medical files has been analysed with individual patient data. This study describes and analyses a wide spectrum of the overall process leading to an OOH-HV after telephone triage and helps understand the factors affecting the quality of reporting in the medical file.

One of the major limitations of our study is its monocentric character, which limits generalizability. Yet, because the electronic patient administration file’s software is the same in the four largest emergency call centres of the Paris area, the reporting in other centres might be similarly affected by factors linked to the system’s ergonomy. Moreover, and because many triage GPs share their activity between several call centres in the Paris area, there might not be a significant centre effect in these results.

Although our study suggests that patient data did not affect the quality of reporting in medical files after telephone triage, we cannot conclude formally because of the low number of variables tested, due to data-mining limitations. As well, we did not take into account the over-sampling fraction of weekends, which may imply selection bias in our adjusted regression analyses.

Current practices do not take into account patients referred to the hospital on their own but only those using ambulances, so the overall number of hospitalized patients after an OOH-HV (8%) may be underestimated. However, in many of the analysed files, the low referral rate suggests that the OOH-HV was the most appropriate decision after triage.

In addition, the quality of reporting may not be linked to the quality of interrogation. Why the quality of reporting remained linked to assistants-related work factors only is unclear. In the absence of a qualitative evaluation of the overall triage process, including communications between assistants and physicians, some factors may remain hidden.

The choice of key indicators was based on the French recommendations for the triage process. Whether their type or number was sufficient is unclear. Some other composite key indicators may be more exhaustive and may lead to other results [31]. For example, we chose to analyse the criterion “suspected diagnosis”, because we believe that it is part of a relevant triage process: when a triage GP formulates and reports one or several diagnostic hypotheses in the medical file, one can reckon that the final decision is adapted to the patient’s need. This choice is discussable in our setting, as the clinical assessment should be left to the mobile GP performing the OOH-HV: the main goal of telephone triage is not to diagnose a given pathology, but to assess the need of the patient. Because of a poor assisted coding process in the administration system at the call centre (ICD-10), we performed systematic data mining and recoded the RFE with the ICPC-2 classification. This could have biased our descriptive results but did not alter the endpoint “diagnostic hypothesis”. Moreover, because our study did not focus on the quality of triage itself, the endpoint “unreported severity” was considered negative when a single criterion was reported. Although this coding was potentially incomplete (“no purpura” present but “no meningeal syndrome” absent in febrile headache), the triage GP may have considered ruling out the severity by orally asking questions that eventually were not reported [20]. Nevertheless, even in this case, GPs may not rule out every severity criterion, because mandatory questions were regularly omitted in other studies.

### Implications for research and practice

First, because in our study, quality in medical reporting was affected by the number of incoming calls and the number of triage assistants and their telephone occupation rate, research in the French setting should first identify factors predicting increases in emergency call centre workloads and lack of staff [21, 32] and model them according to relevant variables, then evaluate the efficiency of corrective measures (recall of triage assistants or physicians). Second, it should identify individual factors associated with altered reporting in terms of triage staff, to identify the settings associated with lower quality of triage and reporting in medical files. Qualitative studies should then be conducted to analyse human factors affecting the process of call handling, medical interrogation and oral transmission between assistants and physicians. Third, because these factors could be educational, research should evaluate the impact of professional education for emergency call centre staff.

Practically, this study could help establish corrective measures in the emergency call centre. First, these measures could consist of targeted increases in the workforce, based on peak workload, especially during public holidays. Second, after feedback from our results, staff in the emergency call centre could undergo practical training in medical-file reporting. Quality criteria need to be better reported, and both the patient’s condition and final decision should be systematically assessed after the OOH-HV. This process is crucial because employees need to be aware of the importance of good traceability, for quality of the healthcare chain (transmission of data to other health stakeholders, or in case of change in assistant or triage staff) or the legal implications of poor reporting.

## Conclusion

After telephone triage at an emergency call centre for patients requiring an OOH-HV, quality in medical reporting seemed to depend on organizational factors only, especially triage assistant-linked factors (number of incoming calls, duration of telephone call, telephone occupation rate).

## Additional file

Additional file 1: Scores of determinants after the two-round RAND/UCLA method. (XLSX 10 kb)

## Data Availability

The datasets generated and/or analysed during the current study are not publicly available due to the fact that they are not translated from French to English, but are available from the corresponding author on reasonable request.

## Abbreviations

CI: Confidence interval
ED: Emergency department
EP: Emergency physician
GP: General practitioner
HAS: Haute autorité de santé
ICD-10: International classification for diseases, 10th version
ICPC-2: International classification for primary care, 2nd version
IQR: Interquartile range
OOH-HV: Out-of-hours home visit
OOH-PC: Out-of-hours primary care
OR: Odds ratio
RAM: Rand-ucla appropriateness method
RFE: Reason for encounter
